# Glycemic variability in relation to oral disposition index in the subjects with different stages of glucose tolerance

**DOI:** 10.1186/1758-5996-5-38

**Published:** 2013-07-23

**Authors:** Tong Chen, Feng Xu, Jian-bin Su, Xue-qin Wang, Jin-feng Chen, Gang Wu, Yan Jin, Xiao-hua Wang

**Affiliations:** 1 Department of Clinical Laboratory, The Second Affiliated Hospital of Nantong University, No. 6 North Hai-er-xiang Road, Chongchuan District, Nantong 226001, China; 2 Department of Endocrinology, The Second Affiliated Hospital of Nantong University, No. 6 North Hai-er-xiang Road, Chongchuan District, Nantong 226001, China

**Keywords:** Glycemic variability, Continuous glucose monitoring, Oral disposition index, Type 2 diabetes

## Abstract

**Background:**

Glucose variability could be an independent risk factor for diabetes complications in addition to average glucose. The deficiency in islet β cell secretion and insulin sensitivity, the two important pathophysiological mechanisms of diabetes, are responsible for glycemic disorders. The oral disposition index evaluated by product of insulin secretion and sensitivity is a useful marker of islet β cell function. The aim of the study is to investigate glycemic variability in relation to oral disposition index in the subjects across a range of glucose tolerance from the normal to overt type 2 diabetes.

**Methods:**

75-g oral glucose tolerance test (OGTT) was performed in total 220 subjects: 47 with normal glucose regulation (NGR), 52 with impaired glucose metabolism (IGM, 8 with isolated impaired fasting glucose [IFG], 18 with isolated impaired glucose tolerance [IGT] and 26 with combined IFG and IGT), 61 screen-diagnosed diabetes by isolated 2-h glucose (DM2h) and 60 newly diagnosed diabetes by both fasting and 2-h glucose (DM). Insulin sensitivity index (Matsuda index, ISI), insulin secretion index (ΔI30/ΔG30), and integrated β cell function measured by the oral disposition index (ΔI30/ΔG30 multiplied by the ISI) were derived from OGTT. All subjects were monitored using the continuous glucose monitoring system for consecutive 72 hours. The multiple parameters of glycemic variability included the standard deviation of blood glucose (SD), mean of blood glucose (MBG), high blood glucose index (HBGI), continuous overlapping net glycemic action calculated every 1 h (CONGA1), mean of daily differences (MODD) and mean amplitude of glycemic excursions (MAGE).

**Results:**

From the NGR to IGM to DM2h to DM group, the respective values of SD (*mean ± SD*) (0.9 ± 0.3, 1.5 ± 0.5, 1.9 ± 0.6 and 2.2 ± 0.6 mmol/), MBG (5.9 ± 0.5, 6.7 ± 0.7, 7.7 ± 1.0 and 8.7 ± 1.5 mmol/L), HGBI [*median(Q1–Q3)*][0.8(0.2–1.2), 2.0(1.2–3.7), 3.8(2.4–5.6) and 6.4(3.2–9.5)], CONGA1 (1.0 ± 0.2, 1.3 ± 0.2, 1.5 ± 0.3 and 1.8 ± 0.4 mmol/L), MODD (0.9 ± 0.3, 1.4 ± 0.4, 1.8 ± 0.7 and 2.1 ± 0.7 mmol/L) and MAGE (2.1 ± 0.6, 3.3 ± 1.0, 4.3 ± 1.4 and 4.8 ± 1.6 mmol/L) were all increased progressively (all *p* < 0.05), while their oral disposition indices [745(546–947), 362(271–475), 203(134–274) and 91(70–139)] were decreased progressively (*p* < 0.05). In addition, SD, MBG, HGBI, CONGA1, MODD and MAGE were all negatively associated with the oral disposition index in each group (all *p* < 0.05) and in the entire data set (*r* = −0.66, –0.66, –0.72, –0.59, –0.61 and −0.65, respectively, *p* < 0.05).

**Conclusions:**

Increased glycemic variability parameters are consistently associated with decreased oral disposition index in subjects across the range of glucose tolerance from the NGR to IGM to DM2h to DM group.

## Background

The deficiency in islet β cell secretion and insulin sensitivity, the two important pathophysiological mechanisms of diabetes, are responsible for disorders of glycemic metabolism [1-3]. Both β cell secretion dysfunction and insulin resistance can be demonstrated long before overt diabetes and may differ in the different stages of glucose tolerance from the normal glucose regulation (NGR) via impaired glucose metabolism (IGM) to early screen-diagnosed diabetes by 2-h glucose (DM2h) to overt diabetes by fasting and 2-h glucose (DM).

Insulin secretion and insulin resistance can be quantified with the hyperglycemic and euglycemic insulin clamp techniques, respectively [4]. However, these techniques are labor intensive and are difficult to apply in clinical practices or in large epidemiological studies. Surrogate measures of insulin secretion and insulin sensitivity have been developed from oral glucose tolerance test (OGTT) [5]. The product of insulin secretion and sensitivity derived from OGTT (ΔI30/ΔG30 × ISI), also termed oral disposition index, is a useful marker of integrated islet β cell function [6]. The oral disposition index as a composite measure may be a better index than either ΔI30/ΔG30 or ISI alone to reflect the notion of declining β cell function and account for glycemic deteriorations from the normal to overt diabetes.

Glucose variability could be an independent risk factor for diabetes complications in addition to average glucose [7-9]. The continuous glucose monitoring (CGM) system can detect glycemic variability in more details than the conventional self-monitoring methods of blood glucose [10,11]. Glycemic variability parameters, which could be calculated from CGM data [12], may differ in the progression from the normal to overt diabetes.

In this study, we investigated the multiple glycemic variability parameters in relation to oral disposition index in the subjects across a range of glucose tolerance from the NGR to IGM to DM2h to overt DM group.

## Methods

### Study subjects

Total 220 subjects were recruited in this study from January 2012 to January 2013: 47 with normal glucose regulation (NGR), 52 with impaired glucose metabolism (IGM, 8 with isolated impaired fasting glucose [IFG], 18 with isolated impaired glucose tolerance [IGT] and 26 with combined IFG and IGT), 61 screen-diagnosed diabetes by isolated 2-h glucose (DM2h) and 60 newly diagnosed diabetes by both fasting and 2-h glucose (DM). The diagnosis of IFG, IGT and type 2 diabetes were based on the ADA diagnostic criteria 2011 [13]. The oral glucose tolerance test (OGTT) screen program was mainly performed in subjects with diabetes risk factors, such as first-degree relative with diabetes, body mass index > 24 kg/m^2^, dyslipidemia, hypertension, et al. Patients with IGM or DM2h were screened and diagnosed by twice 75-g OGTT. Patients with symptomic hyperglycemia and overt diabetes were diagnosed by once 75-g OGTT. Patients have no acute complications, such as diabetic ketoacidosis, or other disorders affecting glucose metabolism, and did not received anti-diabetic drug treatment. The study was approved by the institutional review board of the Second Affiliated Hospital of Nantong University, with written informed consent being obtained from all participants.

### Baseline measurements

Baseline measurements, including height, weight, and blood pressure, were obtained from all subjects in light clothing and without shoes. Body mass index (BMI) was calculated by dividing weight (kg) by height squared (m^2^). Systolic blood pressure (SBP) and diastolic blood pressure (DBP) were taken three times using a sphygmomanometer and then averaged for further analysis.

### β cell function determination

All subjects were examined by the 75-g oral glucose test. Blood samples were taken at 0, 30, 60, 90, and 120 min for the measurement of plasma glucose and insulin concentrations (glucose unit: mmol/L, insulin unit: miu/L). Insulin sensitivity was estimated using the insulin sensitivity index (ISI) of Matsuda and DeFronzo: ISI = 10,000/square root of (Ins0 × Glu0) × (mean glucose × mean insulin during OGTT) [14]. Insulin secretion was estimated by the insulinogenic index calculated from the ratio of increments of serum insulin to glucose measured at 30 min by the follows: ΔI30/ΔG30 = (Ins30-Ins0)/(Glu30-Glu0) [15]. Integrated β cell function was measured by the oral disposition index as the product of Insulin secretion and insulin sensitivity ((ΔI30/ΔG30) × ISI) [6].

### Continuous glucose monitoring (CGM) in subjects

All subjects were monitored by CGM system (Medtronic MiniMed, Northridge, CA 91325, USA) for 72 hours after OGTT. The CGM system sensor was inserted in all patients on day 0 and removed on day 3. Data were downloaded and glucose profiles were evaluated based on the data collected on day 1 and 2. The patients were instructed to input at least four calibration readings per day and the times of key events. During the study, all subjects had standard meals provided by dietary division. The total calorie intake was 30 kcal/kg per day, with 50% carbohydrates, 15% proteins, and 35% fats. The calorie distribution between breakfast, lunch, and dinner was 20%, 40%, and 40%, respectively. Three daily meals were required to consume at time of 6:30 to 7:30, 11:30 to 12:30, and 18:00 to 19:00, respectively.

The parameters of glycemic variability included the standard deviation of blood glucose (SD), mean of daily continuous 24 hours blood glucose (MBG), high blood glucose index (HBGI), continuous overlapping net glycemic action calculated every 1 h (CONGA1), mean of daily differences (MODD) and mean amplitude of glycemic excursions (MAGE) [12]. Continuous overlapping net glycemic action (CONGA) was calculated by determining the difference between values at different set intervals [16], and CONGA1 was calculated every 1 h during the monitoring period. High blood glucose index (HBGI) was used to assess the hyperglycemic risk [17]. The mean of daily differences (MODD) was calculated from the absolute difference between paired continuous glucose monitoring values during two successive 24 hour periods and was used to assess inter-day glycemic variability [18]. The mean amplitude of glycemic excursions (MAGE), which was designed to quantify major swings of glycemia and to exclude minor ones, was used for assessing intra-day glycemic variability in this study [19,20]. It should be noted that MBG is a measure of quality of glycemic control and not specifically variability.

### Laboratory examination

Capillary glucose concentrations were measured with Lifescan Surestep blood glucose meter. Glycosylated hemoglobin A1c (HbA1c) was measured by the high performance liquid chromatography (HPLC) with D-10 hemoglobin Testing Program (Bio-Rad). Serum glucose concentrations were measured by the glucose oxidase method. Serum insulin concentrations were measured by magnetic beads-based enzymatic spectrofluorometric immunoassay with automatic enzyme immunoassay apparatus (AIA360, TOSOH). Total cholesterol (TC), triglyceride (TG), high density lipoprotein cholesterol (HDL-C), and low density lipoprotein cholesterol (LDL-C) were measured with Hitachi Model 7600 Series Automatic Analyzer.

### Statistical analyses

Data analyses were performed using the SPSS16.0 statistical software (SPSS Inc., USA). Continuous variables were expressed as means ± standard deviation (SD) or median (interquartile range) in the case of skewed distributions, and sex ratio were expressed as *n* (%). Natural logarithmic transformations were applied for all non-normally distributed variables.

The validity of the oral disposition index was assessed by demonstrating whether a hyperbolic relationship existed between OGTT-based measures of insulin secretion and insulin sensitivity. Regression analysis was applied to combinations of insulin secretion and insulin sensitivity to determine the regression coefficient β for the following model: ln(insulin secretion) = constant + β × ln(insulin sensitivity). The hyperbolic relationship can be established if estimated β is approximately equal to −1and with 95% confidence interval (CI) excluding 0 [21,22].

The one-way analysis of variance (ANOVA) test were applied to compare differences of continuous variables among groups, and the least significant difference (LSD) test for multiple comparisons was further performed. Chi-squared test was applied to compare sex distribution among groups. Relationships between glycemic variability and oral disposition index were assessed using the Pearson’s correlation test. *p* < 0.05 was considered to be statistically significant.

## Results

### Baseline characteristics of the subjects

As shown in Table 1, age, sex distribution and BMI were comparable among the four groups. The blood pressure (SBP and DBP) in the IGM, DM2h and DM groups were generally higher than those in the NGR group. The lipid metabolic disorders were also observed in the IGM, DM2h and DM groups. The glycosylated hemoglobin A1c (HbA1c) was increased progressively from the NGR to IGM to DM2h to DM group (Table 1).

**Table 1:** Clinical characteristics in subjects with different stages of glucose tolerance

	**NGR**	**IGM**	**DM2h**	**DM**	***P***
***N***	**47**	**52**	**61**	**60**	**–**
Age(year)	45 ± 10	46 ± 13	48 ± 13	44 ± 12	0.093
Female(%)	22(46.8)	21(40.4)	26(51.0)	28(46.7)	0.886
BMI(kg/m^2^)	24.8 ± 3.4	25.2 ± 2.8	24.7 ± 2.5	25.0 ± 4.4	0.182
SBP(mmHg)	119 ± 12	126 ± 13*	125 ± 15*	132 ± 18*	0.001
DBP(mmHg)	75 ± 10	77 ± 9	79 ± 10*	80 ± 8*	0.015
TG(mmol/L)	1.4(0.8–2.2)	1.5(0.9–2.5)	1.9(1.1–3.3)*‡	1.8(1.4–4.0)*‡	0.002
TC(mmol/L)	4.5 ± 1.2	4.7 ± 1.0	5.1 ± 1.3*	5.3 ± 2.0*‡	0.022
HDL-C(mmol/L)	1.4 ± 0.4	1.2 ± 0.3*	1.1 ± 0.3*	1.1 ± 0.3*	0.013
LDL-C(mmol/L)	2.3 ± 0.8	2.5 ± 0.8	2.8 ± 1.0*‡	2.6 ± 0.7*	0.020
HbA1c(%)	5.3 ± 0.5	6.3 ± 1.1*	7.6 ± 1.6*‡	8.9 ± 1.8*‡§	0.000
ISI	168(122–222)	131(97–203)*	108(74–162)*‡	92(67–142)*‡	0.002
ΔI30/ΔG30	4.5(3.7–5.8)	2.6(1.4–4.4)*	1.6(1.1–3.1)*‡	1.0(0.5–1.9)*‡§	0.000
oral disposition index	745(546–947)	362(271–475)*	203(134–274)*‡	91(70–139)*‡§	0.000
SD(mmol/L)	0.9 ± 0.3	1.5 ± 0.5*	1.9 ± 0.6*‡	2.2 ± 0.6*‡§	0.000
MBG(mmol/L)	5.9 ± 0.5	6.7 ± 0.7*	7.7 ± 1.0*‡	8.7 ± 1.5*‡§	0.000
HBGI	0.8(0.2–1.2)	2.0(1.2–3.7)*	3.8(2.4–5.6)*‡	6.4(3.2–9.5)*‡§	0.000
CONGA1(mmol/L)	1.0 ± 0.2	1.3 ± 0.2*	1.5 ± 0.3*‡	1.8 ± 0.4*‡§	0.000
MODD(mmol/L)	0.9 ± 0.3	1.4 ± 0.4*	1.8 ± 0.7*‡	2.1 ± 0.7*‡§	0.000
MAGE(mmol/L)	2.1 ± 0.6	3.3 ± 1.0*	4.3 ± 1.4*‡	4.8 ± 1.6*‡§	0.000

Normally distributed values in the table are given as the mean ± SD, and the non-normally distributed values are given as the median (25% and 75% interquartiles).

*NGR* normal glucose regulation, *IGM* impaired glucose metabolism, *DM2h* screen-diagnosed diabetes by isolated 2-h glucose, *DM* newly diagnosed diabetes by both fasting and 2-h glucose.

*BMI* body mass index, *SBP/DBP* systolic/diastolic blood pressure, *TC* total cholesterol, *TG* triglyceride, *HDL-C* high density lipoprotein cholesterol, *LDL-C* low density lipoprotein cholesterol, *HbA1c* glycosylated hemoglobin A1c, *ISI* insulin sensitivity index, *ΔI30/ΔG30* serum insulin to glucose measured at 30 min, *SD* standard deviation of blood glucose, *MBG* mean of blood glucose, *HBGI* high blood glucose index, *CONGA1* continuous overlapping net glycemic action calculated every 1 h, *MODD* mean of daily differences, *MAGE* mean amplitude of glycemic excursions.

Sex distribution was compared by Chi-squared test; comparison to NGR: *p < 0.05; comparison to IGM: ‡*p* < 0.05; comparison to DM2h: §*p* < 0.05.

### β cell function index derived from OGTT in the subjects

Insulin secretion index (ΔI30/ΔG30) was decreased progressively from the NGR to IGM to DM2h to DM group (*p* < 0.05). Matsuda index (ISI) in the NGR and IGM groups were higher than in DM2h and DM groups (*p* < 0.05), and the DM2h and DM groups had no differences in ISI (*p* > 0.05) (Table 1).

**Figure 1:**
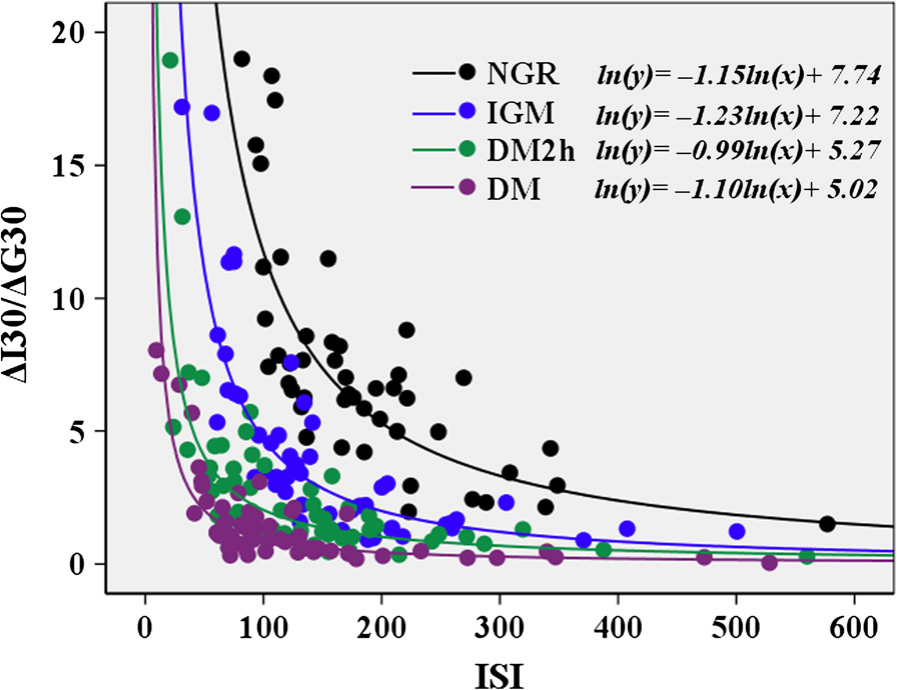
**Hyperbolic relationship between insulin secretion index(ΔI30/ΔG30) and insulin sensitivity index(ISI) in subjects with different stages of glucose tolerance.** NGR: normal glucose regulation; IGM: impaired glucose metabolism; DM2h: screen-diagnosed diabetes by isolated 2-h glucose; DM: newly diagnosed diabetes by both fasting and 2-h glucose.

The oral disposition index was decreased progressively from the NGR to IGM to DM2h to DM group (*p* < 0.01) (Table 1). Figure 1 displays the hyperbolic relationship between insulin secretion and insulin sensitivity for each glucose tolerance group using the product of the ΔI30/ΔG30 and ISI. The hyperbolic curves demonstrated a shift to the left and downward from the NGR to IGM to DM2h to DM. The corrected slopes included −1 for the relationship between ln(ΔI30/ΔG30) and ln(ISI) in the NGR (−1.15 [95% CI −1.39 to −0.91]), IGM (−1.23 [−1.44, –1.03]), DM2h (−0.99 [−1.19, –0.80]) and DM (−1.10 [−1.31, –0.90]). So the validity of the oral disposition index was documented.

### Glycemic variability in the subjects

The multiple glycemic variability parameters from CGM data were shown in Table 1. After comparison within the four groups, SD, MBG, HGBI, CONGA1, MODD and MAGE were all increased progressively from the NGR to IGM to DM2h to DM group (*p* < 0.05).

### Inter-relationships among glycemic variability in the subjects

The Spearman’s correlation coefficients among the parameters of glycemic variability were analyzed to measure whether they have agreements in characterizing glycemic variability (Table 2). There are high degrees of agreement among SD, MBG, HGBI, CONGA1, MODD and MAGE, with almost correlations being significant at the *p* < 0.01 level in each glucose tolerance group and in the entire data set.

**Table 2:** Correlation coefficients among the glycemic variability in subjects with different stages of glucose tolerance

		**MBG**		**CONGA1**		**HBGI**		**MODD**		**MAGE**	
		***r***	***p***	***r***	***p***	***r***	***p***	***r***	***p***	***r***	***p***
NGR	SD	0.47	<0.001	0.63	<0.001	0.71	<0.001	0.76	<0.001	0.83	<0.001
	MBG			0.88	<0.001	0.70	<0.001	0.29	0.048	0.64	<0.001
	CONGA1					0.69	<0.001	0.56	<0.001	0.37	0.011
	HBGI							0.40	0.005	0.64	<0.001
	MODD									0.57	<0.001
IGM	SD	0.66	<0.001	0.65	<0.001	0.95	<0.001	0.87	<0.001	0.85	<0.001
	MBG			0.93	<0.001	0.76	<0.001	0.68	<0.001	0.52	<0.001
	CONGA1					0.71	<0.001	0.74	<0.001	0.56	<0.001
	HBGI							0.84	<0.001	0.68	<0.001
	MODD									0.52	<0.001
DM2h	SD	0.66	<0.001	0.68	<0.001	0.82	<0.001	0.84	<0.001	0.78	<0.001
	MBG			0.92	<0.001	0.76	<0.001	0.58	<0.001	0.48	<0.001
	CONGA1					0.77	<0.001	0.37	0.003	0.55	<0.001
	HBGI							0.70	<0.001	0.55	<0.001
	MODD									0.59	<0.001
DM	SD	0.56	<0.001	0.59	<0.001	0.83	<0.001	0.78	<0.001	0.84	<0.001
	MBG			0.94	<0.001	0.89	<0.001	0.60	<0.001	0.40	<0.001
	CONGA1					0.85	<0.001	0.57	<0.001	0.44	<0.001
	HBGI							0.66	<0.001	0.66	<0.001
	MODD									0.57	<0.001
Total	SD	0.76	<0.001	0.78	<0.001	0.84	<0.001	0.84	<0.001	0.86	<0.001
	MBG			0.96	<0.001	0.80	<0.001	0.75	<0.001	0.68	<0.001
	CONGA1					0.73	<0.001	0.67	<0.001	0.58	<0.001
	HBGI							0.74	<0.001	0.75	<0.001
	MODD									0.74	<0.001

*NGR* normal glucose regulation, *IGM* impaired glucose metabolism, *DM2h* screen-diagnosed diabetes by isolated 2-h glucose, *DM* newly diagnosed diabetes by both fasting and 2-h glucose, *SD* standard deviation of blood glucose, *MBG* mean of blood glucose, *HBGI* high blood glucose index, *CONGA1* continuous overlapping net glycemic action calculated every 1 h, *MODD* mean of daily differences, *MAGE* mean amplitude of glycemic excursions.

In the majority of published studies, the standard deviation (SD) around the mean glucose value was considered as a classical and well-validated index to assess the glycemic variability. The ratios of MAGE, MODD and CONGA1 to SD were also analyzed in the study subjects. There were direct linear proportionalities between MAGE, MODD, CONGA1 and SD for the each glucose tolerance group or the entire data set. The ratios of MAGE, MODD and CONGA1 to SD were 2.24 ± 0.031 (SEM), 0.94 ± 0.014 and 0.89 ± 0.01, respectively, in the entire data set. And the ratios of MAGE/SD, MODD/SD and CONGA1/SD were comparable in the NGR, IGM, DM2h and DM groups (ANOVA *p* = 0.988, *p* = 0.320 and 0.350, respectively) (Table 3).

**Table 3:** The ratios of MAGE, MODD and CONGA1 to SD in subjects with different stages of glucose tolerance

	**NGR**	**IGM**	**DM2h**	**DM**	***P***	**Total**
MAGE/SD	2.26 ± 0.060	2.23 ± 0.060	2.23 ± 0.064	2.25 ± 0.060	0.988	2.24 ± 0.031
MODD/SD	0.99 ± 0.030	0.93 ± 0.026	0.92 ± 0.030	0.92 ± 0.026	0.320	0.94 ± 0.014
CONGA1/SD	0.93 ± 0.02	0.88 ± 0.02	0.89 ± 0.02	0.87 ± 0.03	0.350	0.89 ± 0.01

The ratios in the table are given as the mean ± SEM.

*NGR* normal glucose regulation, *IGM* impaired glucose metabolism, *DM2h* screen-diagnosed diabetes by isolated 2-h glucose, *DM* newly diagnosed diabetes by both fasting and 2-h glucose, *SD* standard deviation of blood glucose, *MBG* mean of blood glucose, *HBGI* high blood glucose index, *CONGA1* continuous overlapping net glycemic action calculated every 1 h, *MODD* mean of daily differences, *MAGE* mean amplitude of glycemic excursions.

### Relationships between glycemic variability and oral disposition index

When the relationships between glycemic variability parameters and oral disposition index were analyzed by Pearson’s correlation test, SD, MBG, HGBI, CONGA1, MODD and MAGE were all negatively associated with oral disposition index in each group (*p* < 0.05) and in the entire data set (*p* < 0.05) (Table 4). These relationships remained significant after adjusting for age, sex, BMI, SBP, DBP, TG, TC, HDLC, LDLC and HbA1c.

**Table 4:** Relationships between glycemic variability and oral disposition index in subjects with different stages of glucose tolerance

	**NGR**		**IGM**		**DM2h**		**DM**		**Total**	
	***r***	***p***	***r***	***p***	***r***	***p***	***r***	***p***	***r***	***p***
SD	−0.29	0.048	−0.38	0.006	−0.36	0.005	−0.45	<0.001	−0.66	<0.001
MBG	−0.56	<0.001	−0.35	0.012	−0.42	0.001	−0.40	0.001	−0.66	<0.001
HBGI	−0.30	0.043	−0.32	0.023	−0.47	<0.001	−0.47	<0.001	−0.72	<0.001
CONGA1	−0.47	0.001	−0.34	0.015	−0.43	0.001	−0.32	0.014	−0.59	<0.001
MODD	−0.33	0.022	−0.48	<0.001	−0.30	0.018	−0.41	0.001	−0.61	<0.001
MAGE	−0.55	<0.001	−0.41	0.003	−0.34	0.007	−0.40	0.002	−0.65	<0.001

HBGI and oral disposition index were ln-transformed.

*NGR* normal glucose regulation, *IGM* impaired glucose metabolism, *DM2h*, screen-diagnosed diabetes by isolated 2-h glucose, *DM* newly diagnosed diabetes by both fasting and 2-h glucose, *SD* standard deviation of blood glucose, *MBG* mean of blood glucose, *HBGI* high blood glucose index, *CONGA1* continuous overlapping net glycemic action calculated every 1 h, *MODD* mean of daily differences, *MAGE* mean amplitude of glycemic excursions.

**Figure 2:**
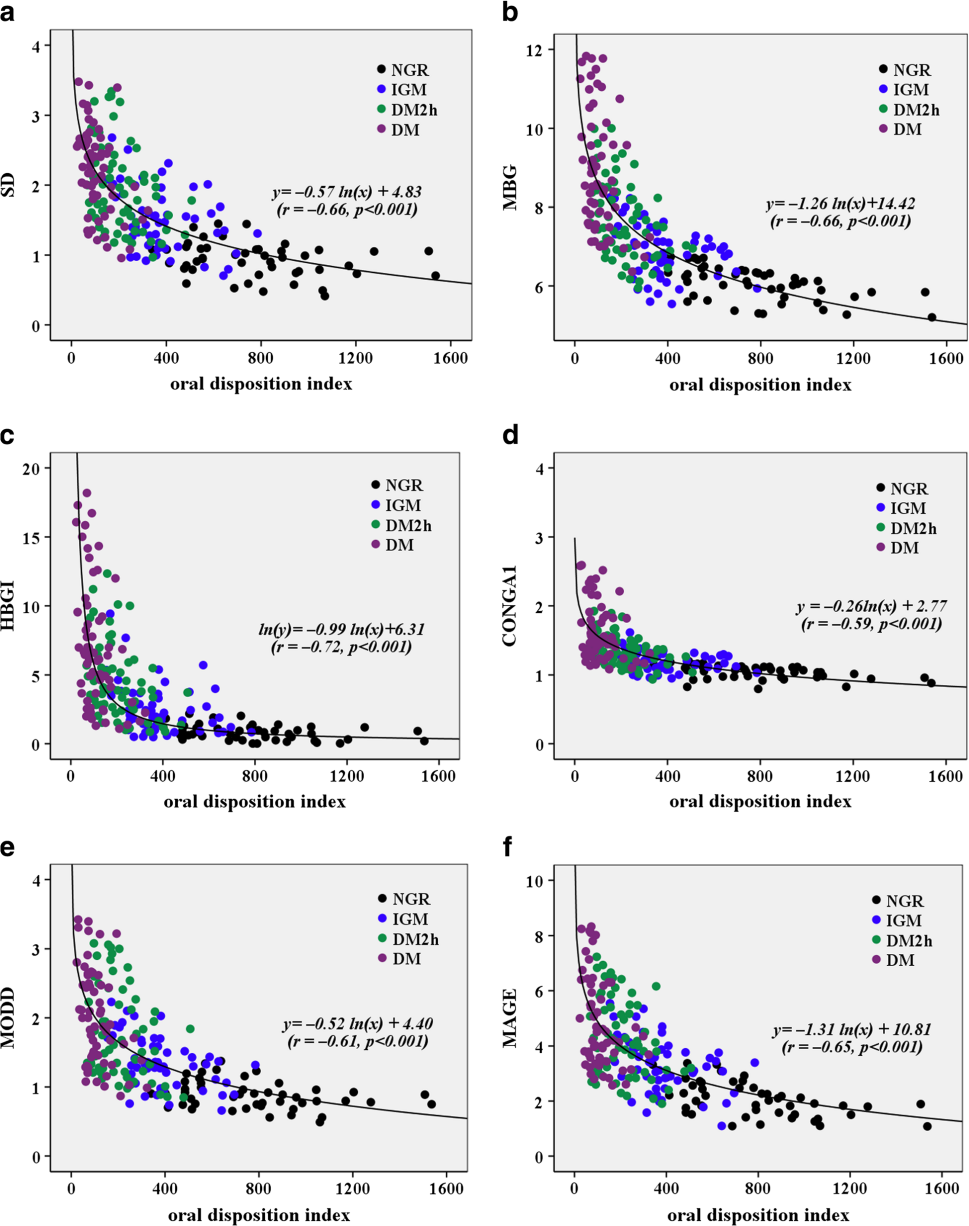
**The overall best-fit lines obtained by nonlinear regression analysis between glycemic variability parameters (a: SD, b: MBG, c: HGBI, d: CONGA1, e: MODD and f: MAGE) and oral disposition index.** NGR: normal glucose regulation; IGM: impaired glucose metabolism; DM2h: screen-diagnosed diabetes by isolated 2-h glucose; DM: newly diagnosed diabetes by both fasting and 2-h glucose. SD: standard deviation of blood glucose; MBG: mean of blood glucose; HBGI: high blood glucose index; CONGA1: continuous overlapping net glycemic action calculated every 1 h; MODD: mean of daily differences; MAGE: mean amplitude of glycemic excursions.

The overall best-fit lines obtained by nonlinear regression analysis (logarithmic regression) between glycemic variability parameters and oral disposition index are presented in the Figure 2a–f. The overall regression line between SD and oral disposition index was y = −0.57ln(x) + 4.83(*r* = −0.66, *p* < 0.001), overall regression line between MBG and oral disposition index was y = −1.26ln(x) + 14.42 (*r* = −0.66, *p* < 0.001), overall regression line between HBGI and oral disposition index was ln(y) = −0.99ln(x) + 6.31 (*r* = −0.72, *p* < 0.001), overall regression line between CONGA1 and oral disposition index was y = −0.26ln(x) + 2.77(*r* = −0.59, *p* < 0.001), overall regression line between MODD and oral disposition index was y = −0.52ln(x) + 4.40 (*r* = −0.61, *p* < 0.001), overall regression line between MAGE and oral disposition index was y = −1.31ln(x) + 10.81(*r* = −0.65, *p* < 0.001). The correlation coefficients were almost in agreements (Figure 2a–f).

## Discussion

The glycemic disorders in diabetes are not solely limited to fasting and postprandial hyperglycemia, but can be extended to the glycemic variability that includes both upward (postprandial glucose increments) and downward (interprandial glucose decrements) changes [23]. Glucose variability could be an independent risk factor for diabetes complications in addition to average glucose [7-9]. Glucose fluctuations are presented not only in diabetes patients but also in normoglycemic and prediabetes subjects [24], and the characteristics of glucose metabolic disorders may differ in the progression from normal to overt diabetes. Our study recruited the subjects across a range of glucose tolerance from the NGR to IGM to DM2h to overt DM group. The results of our study showed glycemic variability parameters, including SD, MBG, HGBI, CONGA1, MODD and MAGE, increased progressively from the NGR to IGM to DM2h to DM group. The results of our study are consistent with those of Wang et al. [25]. Wang et al. showed the diabetes patients had increased postprandial glucose excursion, higher glucose levels overnight and greater inter-day fluctuations compared with the normoglycemic and impaired glucose regulation individuals. Our findings could have a major impact on our understanding of the overall glycemic variability changes from the NGR to IGM to DM2h to overt DM group and how this could influence different complication consequences.

In addition to metabolic differences, other factors such as meals consumed and drug treatment may be partly responsible for the glycemic variability [26]. We employed a fixed meal regimen in the study and recruited diabetes patients (DM2h and DM groups) who had not received anti-diabetic drug treatment thereby controlling for the dietary and drug effect on glycemic variability. Thus, changes of glycemic variability due to metabolic differences from the normal to overt diabetes were well demonstrated in our study.

The inter-relationships among measures of variability were further assessed to weigh whether they have agreement in characterizing glycemic variability. The results documented that the agreements among the measures were at high degree in each glucose tolerance group and in the entire data set. The results were consistent with those from Hill et al. [11] and Kohnert et al. [27]. In the majority of published studies, the SD around the mean glucose value was considered as a classical and well-validated index to assess the glycemic variability [28]. The ratios of MAGE, MODD and CONGA1 to SD were 2.24 ± 0.031 (SEM), 0.94 ± 0.014 and 0.89 ± 0.01, respectively, in the entire data set. The ratios that we have identified fit well with previously reported values of Rodbard et al. [28-30], Fritzsche et al. [31] and Kohnert et al. [27]. The ratio of MAGE/SD was also comparable in NGR, IGM, DM2h and DM groups. And the ratios of MODD/SD and CONGA1/SD were also similar in the four groups. Thus, our findings imply that SD, CONGA1, MODD and MAGE could be used as validated indices to assess the glycemic variability in subjects with different stages of glucose tolerance.

The progression from the NGR via IGM to DM2h to overt diabetes was considered due to the deterioration of insulin secretion and increasing of insulin insensitivity. Insulin secretion index (ΔI30/ΔG30), a surrogate marker of early insulin response, has been validated against gold-standard measures of insulin secretion obtained from intravenous glucose tolerance testing [32]. The decreased insulin secretion capacity plays a definite role in the development of type 2 diabetes [33,34]. Matsuda index, a marker of whole-body insulin sensitivity, is highly correlated with the rate of whole-body glucose disposal during the euglycemic insulin clamp [14]. Kim et al. [6] found that increased risk for type 2 diabetes was evidenced by a lower oral disposition index (ΔI30/ΔG30 × ISI). Our study demonstrated that the oral disposition index declined progressively from the NGR to IGM (IFG/IGT) to early screen-diagnosed diabetes by isolated 2-h post-challenge glucose to overt diabetes by fasting and 2-h glucose. The hyperbolic curves representing the oral disposition index are shifted to the left and downward from the NGR to IGM to DM2h to DM (Figure 1). This shift is also a hallmark of type 2 diabetes pathophysiology and is considered one of the earliest indicators of β-cell dysfunction [35].

After correlation analysis, SD, MBG, HGBI, CONGA 1, MODD and MAGE were all negatively associated with the oral disposition index in the separate group (NGR, IGM, DM2h or DM) and in the entire data set. These findings imply that diabetic treatment aims to improve β cell function may flat glycemic variability.

It should be pointed out that our study has some limitations. The IGM group should be theoretically divided into subgroups with isolated IFG or IGT, but the small sample size of subgroups might make some differences insignificant. We put them into one study group considering that IFG and IGT are intermediate states that exist between normal glucose tolerance and overt diabetes. Another limitation related to oral disposition index is that circulating insulin levels during the OGTT may be affected by other factors apart from β cell function, such as incretin hormones and hepatic extraction. The two factors may limit the degree to which insulin levels during the OGTT can reflect β cell function. But the validity of the oral disposition index had been assessed by demonstrating that the hyperbolic relationship existed between OGTT-based measures of insulin secretion and insulin sensitivity (Figure 1).

## Conclusion

In summary, our study has demonstrated that increased glycemic variability parameters are consistently associated with decreased oral disposition index in the subjects across the range of glucose tolerance from the NGR to IGM to DM2h to DM group, which not only provides important clues for clinics but also forms a strong basis for further investigations of glycemic disorders.

## Abbreviations

NGR: Normal glucose regulation; IGM: Impaired glucose metabolism; DM2h: Screen-diagnosed diabetes by isolated 2-h glucose; DM: Newly diagnosed diabetes by both fasting and 2-h glucose; BMI: Body mass index; SBP/DBP: Systolic/diastolic blood pressure; TC: Total cholesterol; TG: Triglyceride; HDL-C: High density lipoprotein cholesterol; LDL-C: Low density lipoprotein cholesterol; HbA1c: Glycosylated hemoglobin A1c; ISI: Insulin sensitivity index; ΔI30/ΔG30: Serum insulin to glucose measured at 30 min; SD: Standard deviation of blood glucose; MBG: Mean of blood glucose; HBGI: High blood glucose index; CONGA1: Continuous overlapping net glycemic action calculated every 1 h; MODD: Mean of daily differences; MAGE: Mean amplitude of glycemic excursions.

## Competing interests

The authors have no competing interests to declare.

## Authors’ contributions

TC and FX participated in the design of the study, data collection, analysis of the data, drafting of the manuscript. JS conceived of the study, participated in its design and revised the manuscript. XW participated in analysis of the data and revised the manuscript. JC, GW, YJ and XW participated in data collection. All authors read and approved the final manuscript.
